# T251. THE STUDY OF QUALITY OF LIFE AND A GLOBAL FUNCTIONING FOR THE SCHIZOPHRENIA PATIENTS IN COMMUNITY BY THEIR RESIDENTIAL ENVIRONMENT

**DOI:** 10.1093/schbul/sby016.527

**Published:** 2018-04-01

**Authors:** Sang-Yeol Lee, Young-Joon Kwon, Bo-Hyun Yoon, Kwanghun Lee, Moon Doo Kim, Beomwoo Nam, Sung-Yong Park, Eunsung Lim

**Affiliations:** 1 Wonkwnag University School of Medicine and Hospital; 2 Soonchunhyang University Chunan Hospital; 3 Naju National Hospital; 4 College of Medicine, Dongguk University; 5 School of Medicine, Jeju National University; 6 School of Medicine, Konkuk University; 7 Keyo Hospital; 8 Shinsegae Hospital

## Abstract

**Background:**

Mental health is so deeply related to our residential environment that the difficulties of residence could worsen it for schizophrenia patients. Moreover, patients with schizophrenia might induce severe residential problems such as poverty, discrimination, failure to education, frequent migration, homeless, and etc. This study investigated the quality of life and a global functioning for schizophrenia patients in community by their residential environment.

**Methods:**

Total 648 patients with schizophrenia living in Jeollabukdo(province) were tested demographic and clinical characteristics. Housing and residential satisfaction were measured by the questionnaires established by Ministry of Land, Transport and Maritime Affairs and modified for this study. Psychiatric and psychological variables were assessed by Global Assessment Function (GAF) and World Health Organization Quality of Life Assessment Instrument Brief Form (WHOQOL - BREF). Correlations among variables were analyzed using frequency analysis and Pearson’s product moment correlation coefficient.

**Results:**

As the results of correlations between quality of life and housing satisfaction, correlations were shown at a global quality of life (r=0.312, p<.01), physical health (r=0.227, p<.01), psychological domain (r=0.215, p<.01), social relation domain (r=0.170, p<.01), and environmental domain (r=0.372, p<.01). For the correlation between quality of life and residential area satisfaction, a general quality of life (r=0.307, p<.01), physical health (r=0.242, p<.01), psychological domain (r=0.243, p<.01), social relation domain (r=0.169, p<.01), and environmental domain (r=0.306, p<.01) were correlated.

**Discussion:**

The correlations among residential environment, quality of life, and a global functioning were significant. Consequently, it is necessary for the government policy that can improve housing and residential environment for the mentally disordered and ultimately contribute to enhance their welfare.

